# The mediating effect of internet addiction and the moderating effect of physical activity on the relationship between alexithymia and depression

**DOI:** 10.1038/s41598-024-60326-w

**Published:** 2024-04-29

**Authors:** Yang Liu, Liangfan Duan, Qingxin Shen, Yuanyuan Ma, Yiyi Chen, Lei Xu, Yawen Wu, Tiancheng Zhang

**Affiliations:** 1 School of Sports Science, Jishou University, Jishou, China; 2 Institute of Physical Education, Shanxi University of Finance and Economics, Taiyuan, China

**Keywords:** Alexithymia, Depression, Internet addiction, Physical activity, College students, Psychology and behaviour, Epidemiology

## Abstract

There is a certain relationship between alexithymia and depression, but further investigation is needed to explore their underlying mechanisms. The aims of this study was to explore the mediating role of internet addiction between alexithymia and depression and the moderating role of physical activity. A total of 594 valid responses were included in the analysis, with a mean age of 18.72 years (SD = 1.09). The sample comprised 250 males (42.09%) and 344 females (57.91%). These responses were utilized for descriptive analysis, correlation analysis, regression analysis, and the development of mediation and moderation models. Alexithymia showed positive correlations with depression and internet addiction, and physical activity was negatively correlated with internet addiction and depression. Internet addiction partially mediated the relationship between alexithymia and depression, while physical activity weakened the association between internet addiction and depression, acting as a moderator. Our findings suggest that excessive Internet engagement may mediate the relationship between alexithymia and depression as an emotional regulatory coping strategy, and that physical activity attenuates the predictive effect of Internet addiction on depression.

## Introduction

Alexithymia, often manifested as a multidimensional impairment in recognizing, understanding, and describing emotions^1,2^, is a stable personality trait. Individuals with higher levels of alexithymia experience increasing difficulties in establishing and maintaining interpersonal relationships, perceive less social support, and exhibit lower levels of social skills^3^. Upon transitioning to university, individuals confront a plethora of unknowns and challenges^4^. Particularly within the context of China, the transition to university presents them with more flexible schedules and expanded social circles, with "adaptation" emerging as one of their foremost hurdles due to the characteristic of alexithymia^5^. Consequently, due to impairments in recognizing and responding to emotions, they struggle to develop healthy or intimate social relationships^6,7^, leading to emotional distress and discomfort^8,9^. Individuals with alexithymia inevitably face difficulties in social interactions and maintaining emotional connections due to its inherent characteristics, and it is often associated with other psychological disorders^10^, such as social anxiety^11^, substance abuse^12^, depression^13,14^, eating disorders^15^, and non-suicidal self-injury^16,17^. Hence, comprehending the relationship between alexithymia in university students and other psychological disorders, along with their underlying physiological and psychological mechanisms, is crucial for promoting the healthy development of individuals with alexithymia or predisposition to alexithymia.

Previous studies have found a high correlation between alexithymia and depression in individuals^14,18–20^. This high correlation between alexithymia and depression is not only present in clinical depression populations^21^, but also in non-clinical populations^13^, and similar results have been obtained in studies of populations with other diseases or psychological disorders^14,22,23^. Evidence of a high correlation between alexithymia and depression exists in various populations^20^. Furthermore, depression mediates the relationship between alexithymia and other risk behaviors in individuals^24^, even completely mediating it^25^. Therefore, based on the above review, we hypothesize that there is a positive correlation between alexithymia and depression in college students.

Interpersonal relationships are challenged due to impaired recognition and response to emotions^6,7^ and often fall into emotional distress^8,9^. Hence, individuals with alexithymia attempt to regulate their emotions through compulsive behaviors^26,27^. The internet provides them with an ideal avenue due to its anonymity, convenience of remote interaction^28^, and absence of face-to-face observation^29^, which may mitigate the deficits in understanding and identifying others' emotions associated with alexithymia while fulfilling the need for social interaction. Consequently, individuals with alexithymia may exhibit excessive dependence on and usage of the internet, leading to the development of internet addiction. Internet addiction refers to the pathological use of the Internet by individuals, leading to adverse consequences in personal, social, and occupational life^30^. Based on detection rates of internet addiction among young people in China, it has been found that internet addiction gradually increases with age and grade, reaching 7.7% among high school students^31^, and even exceeding half among college students^32^. Research has found a significant positive correlation between alexithymia and internet addiction in college students^33,34^, and alexithymia significantly predicts internet addiction in college students^35–37^. This predictive effect also exists in studies focusing on a single gender^38^. Additionally, in a large sample study of Chinese college students, a significant relationship between internet addiction and depression was found^39^, and internet addiction significantly predicted subsequent levels of depression in college students^40^. This relationship also exists in other countries and regions^41–44^. Based on the above review, we can establish the second hypothesis of this study, which is that internet addiction plays a mediating role in the relationship between alexithymia and depression in college students.

Research has indicated that dysfunction of the hypothalamic–pituitary–adrenal (HPA) axis is prevalent among individuals with internet addiction^45,46^. Dysfunction of the HPA axis serves as a significant indicator of depression^47^ and is also a crucial predictive factor for depression^48^. Therefore, regulating the functionality of the HPA axis naturally emerges as a potential pathway for alleviating depression^49^. Studies have shown that physical activity can reduce cortisol levels and promote the development of HPA axis function^50,51^. Hence, physical activity may decrease the strong correlation between internet addiction and depression. Research has found that compared to non-internet addicted college students, internet addicted college students have higher levels of depression and lower levels of physical activity^52^. Physical activity can mitigate internet addiction by modulating the neurobiology of the central and autonomic nervous systems^53^, thereby significantly reducing depression levels among internet-addicted college students^54^, and this evidence is also supported by retrospective studies^55^. Therefore, we make the final hypothesis of this study that physical activity can regulate the relationship between internet addiction and depression in college students.

**Figure 1:**
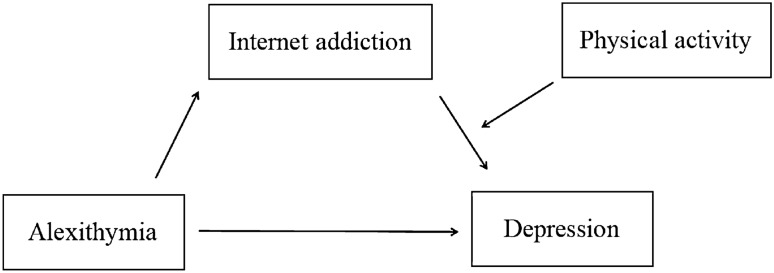
Hypothesized model.

In conclusion, the relationship between alexithymia and depression in college students has been widely reported, but little is known about other psychological factors between the two, including the mediating role of internet addiction and the moderating role of physical activity. This study will use internet addiction as a mediating factor between alexithymia and depression, and physical activity to moderate the relationship between internet addiction and depression, further enriching the underlying psychological mechanisms between alexithymia and depression. A theoretical model diagram is constructed (see Fig. 1).

## Methods

### Participants

The present study was conducted in October 2023 at two universities in the western part of Hunan Province, China. Prior to the commencement of the research, approval was obtained from the Biomedical Ethics Committee of Jishou University (Grant number: JSDX-2023-0034). During the survey, our staff first communicated with the leading teachers of the college students and obtained approval. Subsequently, on a class-by-class basis, the investigators delivered presentations to all participants, informing them of the main content of the survey, the anonymity and confidentiality of the data, their right to freely withdraw, and the disposition of the results. After obtaining informed consent from all individuals, electronic questionnaires were distributed, and participants could complete the questionnaire in its entirety within 20 min. Ultimately, a total of 676 college students completed the survey. We confirm that all the experiment is in accordance with the relevant guidelines and regulations such as the declaration of Helsinki. After screening for insufficient response times (instances where participants completed the questionnaire in an unreasonably short amount of time, suggesting potential rushed or careless responses) and regular patterned responses (responses that exhibit a consistent and predictable pattern, possibly indicating a lack of genuine engagement or random guessing), valid data from 594 participants (250 male, 344 female; 225 non-left-behind, 369 left-behind) were obtained, with an average age of 18.72 years (SD = 1.09).

### Measures

#### Alexithymia

The Toronto Alexithymia Scale (TAS-20) was used to assess the level of alexithymia^2,56^. The scale consists of 20 items, and a Likert 5-point scoring system is used to evaluate the level of alexithymia, ranging from 1 (completely inconsistent) to 5 (completely consistent). Except for the five reverse-scored items (4, 5, 10, 18, and 19), which are scored in reverse, all other items are scored between 1 and 5 points. The sum of all item scores represents the total score of alexithymia, with higher scores indicating more severe levels of alexithymia. In this study, Cronbach's α for the sample was 0.832.

#### Depression

The Chinese version of the Depression Anxiety Stress Scale (DASS-21)^57,58^ was utilized to measure depression. This study employed the depression subscale of the DASS-21, which comprises 7 items and utilizes a Likert 4-point scoring system, ranging from 1 (strongly disagree) to 4 (strongly agree), to assess depression. Each item is scored between 1 and 5 points, and the sum of all item scores represents the total depression score, with higher scores indicating more severe levels of depression. In this study, the Cronbach's α for the sample was 0.899.

#### Internet addiction

The internet addiction level was measured using the Internet Addiction Test (IAT) developed by Wei^59,60^. The questionnaire consists of 8 items and utilizes a Likert 5-point scoring system, ranging from 1 (strongly disagree) to 5 (strongly agree), to evaluate internet addiction. Each item is scored between 1 and 5 points, and the sum of all item scores represents the total internet addiction score, with higher scores indicating more severe levels of internet addiction. In this study, the Cronbach’s α for the sample was 0.876.

#### Physical activity

The physical activity level was measured using the Physical Activity Scale developed by Liang Deqing^61,62^. The scale consists of 3 items, including exercise intensity, duration, and frequency. Each item has 5 different levels, with scores ranging from 1 to 5 for intensity and frequency, and scores ranging from 0 to 4 for duration. The physical activity score is derived by multiplying the scores of the three items, with higher scores indicating higher levels of physical activity. The current sample's Cronbach's alpha for the scale is 0.654.

#### Covariates

Taking into account the influence of demographic variables on the outcome analysis, such as gender and age^35,63^, we controlled for these variables during the analysis. The gender was coded as 1 for male and 2 for female.

### Statistical analyses

In our study, all statistical analyses were conducted using SPSS 26.0 software. Firstly, a method bias test was performed to explore potential biases associated with the use of self-report questionnaires. Subsequently, descriptive statistics and correlation analysis were conducted to describe the demographic characteristics of the participants and the main variables of interest. Prior to further analysis, standardization was applied to the data of the main variables. Finally, to test our hypotheses, we employed the PROCESS macro plugin in SPSS (Model 14) to examine the relationships among alexithymia, depression in college students, the mediating role of internet addiction, and the moderating effect of physical activity^64^. In this process, we utilized 5000 bootstrap resampling iterations to assess model fit and estimate 95% confidence intervals, considering a relationship as significant when the 95% confidence interval did not include zero. Throughout the analysis, gender and age were included as covariates for control analysis.

### Ethics approval and consent to participate

The study was approved by the Biomedicine Ethics Committee of Jishou University before the initiation of the project (Grant number: JSDX-2023-0034). And informed consent was obtained from the participants before starting the program.

## Results

### Harman’s single factor test

The common method bias was examined using Harman's single factor test. The analysis results indicated that among the factors with eigenvalues greater than 1, only two factors met this criterion. Without conducting principal component factor rotation, the first factor accounted for 30.20% of the variance, which was lower than the recommended threshold of 40%^65^. Therefore, based on the analysis results, there was no significant evidence of common method bias in this study.

### Descriptive data and Correlational analyses

Table 1 presents the Pearson correlation data among the variables. Alexithymia is significantly positively correlated with internet addiction (r = 0.413, *p* < 0.001) and depression (r = 0.523, *p* < 0.001) in college students, and significantly negatively correlated with physical activity (r = − 0.255, *p* < 0.001). Internet addiction is significantly positively correlated with depression (r = 0.384, *p* < 0.001) and significantly negatively correlated with physical activity (r = − 0.087, *p* < 0.05) in college students. Physical activity is significantly negatively correlated with depression in college students (r = − 0.269, *p* < 0.001).

**Table 1:** Pearson correlation matrix between relevant variables.

	Mean	SD	1	2	3	4	5
1. Gender	–	–	–				
2. Age	18.72	1.09	− 0.260***	–			
3. Alexithymia	52.23	10.58	0.064	− 0.036	–		
4. Internet addiction	21.51	6.77	0.133**	− 0.005	0.413***	–	
5. Depression	12.31	4.21	0.002	0.070	0.523***	0.384***	–
6. Physical activity	21.73	21.45	− 0.366***	0.300***	− 0.255***	− 0.087*	− 0.269***

*p < 0.05; **p < 0.01; ***p < 0.001.

### Moderated and mediation analysis

**Figure 2:**
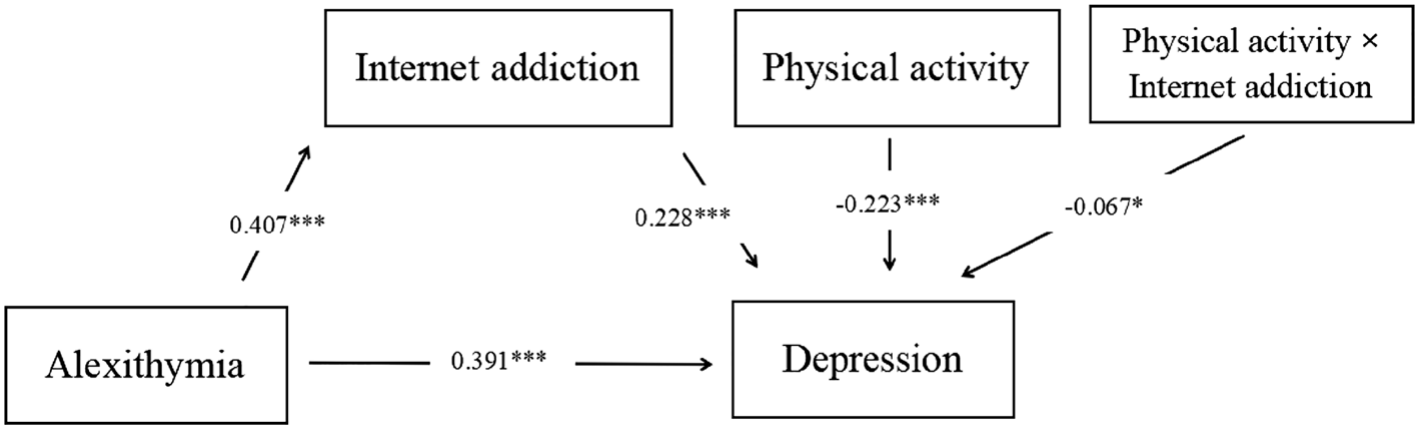
Hypothesized model, *p < 0.05; ***p < 0.001.

**Figure 3:**
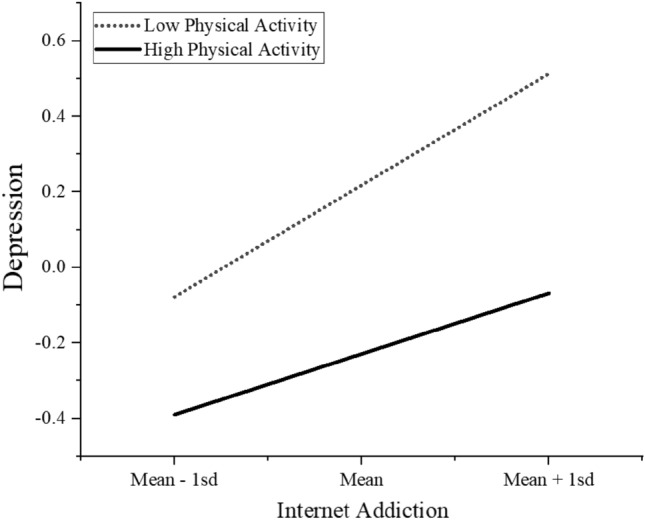
Moderating effect of physical activity on Internet addiction and depression in college students.

After including mediating and moderating variables, as well as controlling for covariates, alexithymia still significantly and positively predicts the level of depression in college students (β = 0.391, SE = 0.038, *p* < 0.001). Furthermore, in the mediation analysis, alexithymia significantly and positively predicts internet addiction in college students (β = 0.407, SE = 0.037, *p* < 0.001), and internet addiction acts as a mediator between alexithymia and the level of depression in college students (β = 0.228, SE = 0.037, *p* < 0.001). In the moderation analysis, physical activity negatively predicts depression in college students (β = − 0.223, SE = 0.038, *p* < 0.001), and the interaction term between internet addiction and physical activity also significantly and negatively predicts depression in college students (β = − 0.067, SE = 0.031, *p* < 0.05). Please refer to Table 2, Figs. 2 and 3 for more details.

**Table 2:** Moderated and mediation analysis.

	Internet addiction			Depression		
	β	SE	t	β	SE	t
Gender	0.239	0.078	3.056**	− 0.206	0.074	− 2.801**
Age	0.037	0.035	1.056	0.110	0.032	3.394***
Alexithymia	0.407	0.037	10.91***	0.391	0.038	10.414***
Internet addiction (A)				0.228	0.037	6.199***
Physical activity (B)				− 0.223	0.038	− 5.922***
A × B				− 0.067	0.031	− 2.192*
R^2^	0.183			0.361		
F	44.176***			55.323***		

*p < 0.05; **p < 0.01; ***p < 0.001.

## Discussion

This study discusses the interrelationships between alexithymia, internet addiction, depression, and physical activity among college students. The results revealed positive correlations between alexithymia, internet addiction, and depression, as well as negative correlations between these variables and physical activity, all of which reached significance levels. After controlling for demographic variables, internet addiction was found to mediate the relationship between alexithymia and depression among college students, while physical activity played a moderating role in the relationship between internet addiction and depression.

Our study found a significant correlation between alexithymia and depression among college students, which is consistent with previous research^20,25^. Alexithymia, characterized by difficulties in recognizing and understanding emotions^66^, has been linked to impaired emotional regulation and an increased risk of depression^67–69^. Furthermore, a longitudinal study found fluctuating scores on the depression scale in conjunction with scores on the alexithymia scale^14^. Additionally, individuals with alexithymia may receive less support^70^, leading to reduced social support and higher levels of depression^71^. Therefore, our results confirm our first hypothesis that there is a significant positive correlation between alexithymia and depression among college students, with alexithymia significantly predicting depression.

Furthermore, this study found that internet addiction plays a mediating role between alexithymia and depression among college students. As previously mentioned, the difficulty of alexithymic individuals in recognizing and understanding emotions leads to difficulties in their interactions with others in the real world^72^. Consequently, they tend to choose to escape the real world and seek social satisfaction in the online world^10^. Compensatory Internet use theory suggests^73^ that negative social relationships and emotions drive individuals to escape into the online world as a coping mechanism, satisfying not only their social needs but also addressing negative emotions. In an era where smartphones and the internet are easily accessible, they find it easier to enter the virtual world. However, this way of coping cannot replace the real world and only leads to the formation and exacerbation of internet addiction. There is also a strong relationship between internet addiction and depression^40^, and longitudinal studies have found that they can predict each other^40,74^. Therefore, based on the evidence mentioned above, our second hypothesis that internet addiction plays a mediating role between alexithymia and students is supported.

Moreover, this study found that physical activity has a moderating effect on the relationship between internet addiction and depression among college students. Loneliness, depression, and sensitivity to interpersonal relationships are significant characteristics of individuals with internet addiction^75^, which, combined with the characteristics of alexithymia, further increases their dependence on the internet^76^. However, previous research has found that exercise can increase levels of neurotrophic factors, cortisol, and neurotransmitters, promote the development of the nervous system, and inhibit reward impulsivity^53^. Additionally, long-term physical exercise can significantly reduce the level of internet addiction and depression among college students, improve sleep quality, and balance the sympathetic and parasympathetic nervous system functions^54^. Moreover, group sports activities are enjoyable and involve social interactions, which may further weaken the depressive emotions caused by internet addiction among college students^55^. Therefore, physical activity can regulate the relationship between internet addiction and depression among college students, confirming our last hypothesis.

This study has certain strengths. It is the first to discuss internet addiction as a mediating factor between alexithymia and depression among college students, and it demonstrates that physical activity can moderate the impact of internet addiction on depression. Hence, while examining the relationship between individual alexithymia and depression, it becomes imperative to assess the presence of internet addiction or other behavioral addictions, as they may exacerbate levels of depression. Concurrently, in light of established patterns of internet addiction behavior, encouraging and leading individuals to actively engage in physical activities is advocated. This not only fosters interaction^77^, learning, and the establishment of positive social relationships during physical activity but also promotes physical and mental well-being^78,79^, potentially reducing depression levels holistically. However, the study also has limitations. Firstly, it is based on cross-sectional data, which may challenge the strength of causal relationships. Future research could use longitudinal data to explain the causal relationships between variables. Secondly, all the main data are self-reported, which may have subjective biases. Future research could combine subjective and objective data to improve the credibility of the evidence. Lastly, this study was conducted based on convenience sampling, which may introduce regional differences. Future research could conduct cross-regional studies.

## Conclusion

This study reveals the relationship between alexithymia and depression among college students, the mediating role of internet addiction between the two, and the moderating effect of physical activity on the relationship between internet addiction and depression. It further enhances our understanding of the relationships and underlying mechanisms among these variables, highlighting the potential roles of these factors in psychological interventions for college students with alexithymia.

## Data Availability

The datasets generated and/or analysed during the current study are not publicly available due (our experimental team's policy) but are available from the corresponding author on reasonable request.
